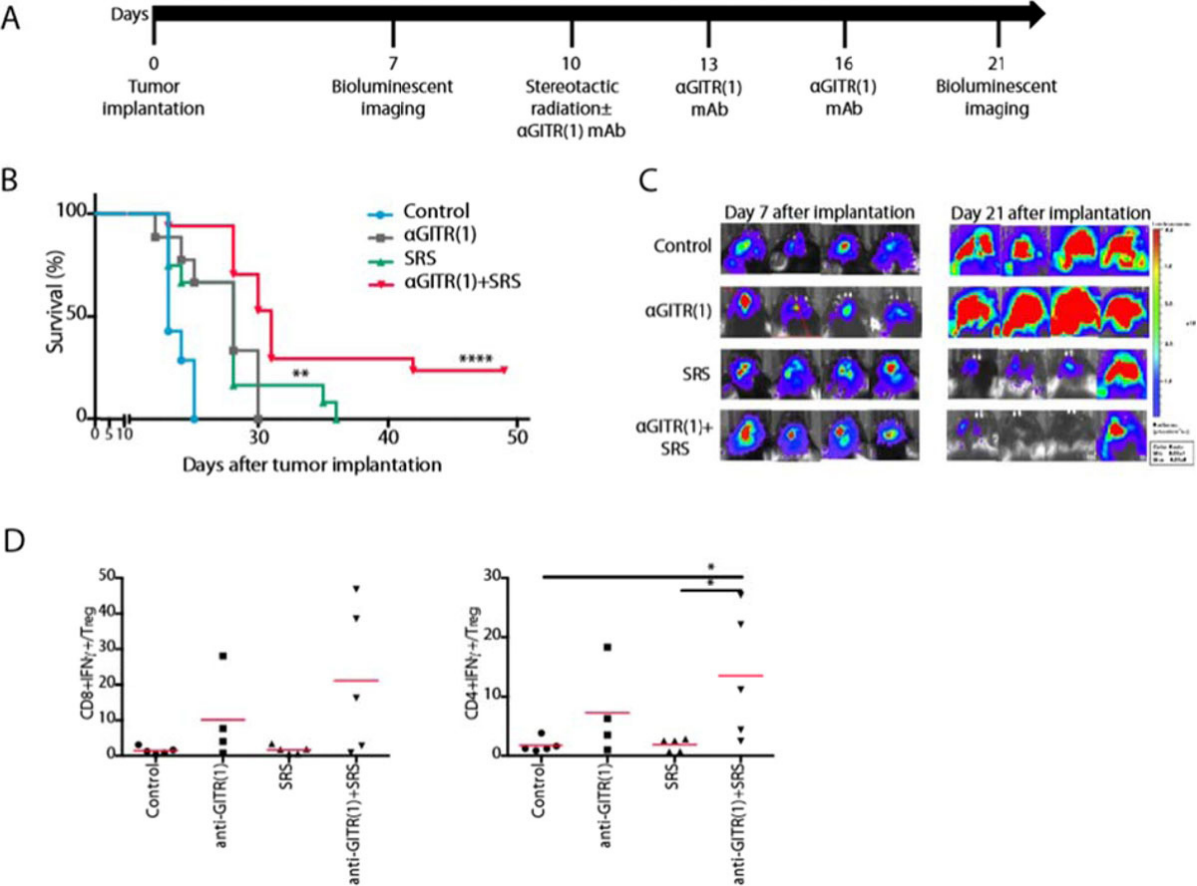# Agonist anti-GITR monoclonal antibody and stereotactic radiation induce immune-mediated survival advantage in murine intracranial glioma

**DOI:** 10.1186/2051-1426-3-S2-P194

**Published:** 2015-11-04

**Authors:** Mira Patel, Jennifer Kim, Debebe Theodros, Christopher Jackson, Ada Tam, Esteban Velarde, Betty Tyler, Xiaobu Ye, Henry Brem, Mark Selby, Charles Drake, Drew Pardoll, Michael Lim

**Affiliations:** 1 Johns Hopkins University School of Medicine, Baltimore, MD, USA; 2 The Johns Hopkins University School of Medicine, Baltimore, MD, USA; 3 The Johns Hopkins University Department of Neurosurgery, Baltimore, MD, USA; 4 The Johns Hopkins University Department of Immunology, Baltimore, MD, USA; 5 The Johns Hopkins University Department of Radiation Oncology, Baltimore, MD, USA; 6 Bristol-Myers Squibb, Baltimore, MD, USA; 7 The Johns Hopkins University Departments of Oncology and Cancer Immunology, Baltimore, MD, USA

## Background

Glioblastoma (GBM) is a poorly immunogenic neoplasm treated with local radiation. Despite the standard of care, median survival remains low. Immunotherapy has synergized with stereotactic radiosurgery (SRS) in murine GBM, as radiation promotes a pro-inflammatory tumor microenvironment amenable to the anti-tumor effects of immune modulation. Glucocorticoid-induced tumor necrosis factor receptor (GITR) is a co-stimulatory receptor expressed constitutively on regulatory T cells and inducibly on effector T cells. We tested the hypothesis that anti-GITR monoclonal antibody (mAb) and SRS combination therapy would confer immune-mediated survival benefit in murine glioma.

## Methods

Mice were implanted with GL261-luc murine glioma cells and began SRS and anti-GITR IgG1 treatment after 10 days. Mice were randomized to four treatment groups: control, SRS only, anti-GITR only, anti-GITR+SRS. SRS was delivered to the tumor in one fraction; mice were given mAb thrice i.p. Mice were euthanized on day 21 to analyze the immunologic profile of tumor, spleen, and tumor draining lymph nodes.

## Results

Anti-GITR mAb plus SRS conferred significantly improved survival over either treatment alone (p < .0001, cure rate 24%). The increased survival required CD4+T cells but not CD8+T cells or regulatory T cells (Tregs). There was elevated intratumoral CD4+ effector-cell infiltration (CD4+/Foxp3-/IFNγ+) relative to Treg infiltration (CD4+/Foxp3+) at day 21 in mice treated with anti-GITR+SRS, and significantly elevated IFNγ and IL-2 production by CD4+T cells and elevated IFNγ and TNFα production by CD8+T cells. Intratumoral mononuclear cells demonstrated increased mRNA expression of pro-inflammatory M1 markers and decreased expression of immunosuppressive M2 markers.

## Conclusions

In all, anti-GITR mAb synergizes with SRS to significantly prolong survival in murine orthotopic glioma in a potentially CD4+ Th1-dominant anti-tumor mechanism with M1 polarization. These findings provide preclinical evidence for the use of anti-GITR IgG1 non-depleting antibodies alongside SRS in human GBM.

**Figure 1:**